# Effects of cyclic nucleotides on midgut infections and maturation of *T. b. brucei *in *G. m. morsitans*

**DOI:** 10.1186/1756-3305-1-5

**Published:** 2008-03-14

**Authors:** Ewan T MacLeod, Ian Maudlin, Susan C Welburn

**Affiliations:** 1 Centre for Infectious Diseases, The University of Edinburgh, Edinburgh, EH25 9RG, UK

## Abstract

Cyclic nucleotide signalling through cyclic adenosine monophosphate (cAMP) is thought to play an important role in the transformation of the long slender (dividing) form to the short-stumpy (arrested) form in the mammalian bloodstream but the role of cyclic nucleotides in the tsetse-based part of the trypanosome life cycle is unknown. In a series of *in vivo *experiments, it was found that cyclic guanosine monophosphate (cGMP) but not cAMP could induce significantly higher rates of midgut infection in tsetse. Continuous feeding of either cGMP or cAMP to tsetse had no effect on rates of maturation of established midgut infections suggesting that these two parts of the life cycle in tsetse are not linked.

## Findings

The short stumpy form of the trypanosome is thought to be pre-adapted to life in the tsetse fly, the long slender form maturing into the short stumpy form once a certain density of infection is reached in the mammalian host [1]. Similar processes happen in the tsetse fly with trypanosomes going through several transformations, starting in the midgut of the fly as bloodstream forms, they transform to procyclic forms before terminal differentiation into mammalian infective forms in the salivary glands (*Trypanosoma brucei *s.l.) or mouthparts (*Trypanosoma congolense*). A link between cAMP and cell cycle signalling in the trypanosome life-cycle was suggested by addition of cAMP analogues *in vitro *which promoted the long slender dividing stage to form non-dividing short stumpy forms [2].

In the present work *Glossina morsitans morsitans *were infected with *T. b. brucei *(stock Buteba 135, see MacLeod *et al*. [3]) on the day following the day of emergence from the puparium. Infective feeds were given *in vitro *using thawed stabilates of trypanosomes suspended in defibrinated ovine blood. To examine the effects of cAMP and cGMP on midgut infection establishment, flies received to a final concentration 1, 10 or 100 μM 8-Br-cGMP or 8-Br-cAMP (Sigma, UK) with their infective bloodmeal (cyclic nucleotides were dissolved in saline then added to the bloodmeal while control flies received saline only).

To examine the effect of 8-Br-cGMP post-infection 100 μM 8-Br-cGMP was added to the bloodmeal 48, 72, 96 or 120 h post infection. Flies which did not take the infective or supplemented feed were removed from the experiment.

To examine the effects of cyclic nucleotides on the maturation of trypanosomes, all flies received a bloodmeal containing 100 μM 8-Br-cGMP and were then either fed 100 μM 8-cAMP or 100 μM 8-Br-cGMP from the second feed onwards. Flies which did not take the infective feed were removed from the experiment.

Following infection, flies were maintained at 25°C and 70% relative humidity and fed on defibrinated ovine blood through an artificial membrane. To determine rates of establishment, flies were dissected 10 d post-infection (or 10 d post-treatment) and midguts were examined for the presence of trypanosomes by phase-contrast microscopy (X400). To determine rates of maturation, flies were dissected 28 d post-infection and midguts and salivary glands were examined for the presence of trypanosomes by phase-contrast microscopy (X400).

Generalised linear models with binomial errors were used to examine the proportion of flies with midgut infections (number of midgut infections/total number of flies dissected) or proportion of midgut infections maturing into mature infections (transmission of infectivity – TI: number of salivary gland infections/number of midgut infections) when compared to control flies (see MacLeod *et al*. [4]).

**Figure 1:**
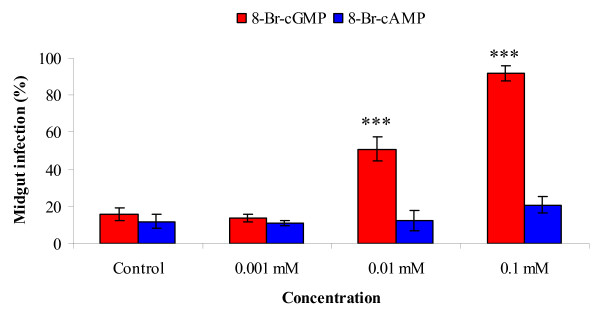
Effect of 8-Br-cGMP or 8-Br-AMP on midgut infections of *T. b. brucei *in male *G. m. morsitans*. Flies were infected at their first feed, the bloodmeal containing 8-Br-cGMP or 8-Br-AMP dissected 10 days later and midguts examined for trypanosome presence by microscopy. Control flies were received saline in their bloodmeal. Data presented as the mean S.E.M. from three experiments. Significance: *** *p *< 0.001 versus the corresponding control value.

The effects of 8-Br-cGMP or 8-Br-cAMP on trypanosome midgut infection rates in male *G. m. morsitans *are shown in Figure 1. Addition of 8-Br-cGMP to the bloodmeal at concentrations of 10 or 100 μM significantly (*p *< 0.001) increased midgut infection rates from 16% control (number of flies dissected, n = 95) to 51% (n = 106) and 92% (n = 86) respectively. Addition of 1 μM 8-Br-cGMP resulted in midgut infection rates of 13% (n = 97) and was not significantly different (*p *= 0.817) from the control. There was no significant difference in infection rates between male and female flies fed the same concentrations of 8-Br-cGMP (data not shown).

8-Br-cAMP had no significant (*p *= 0.131) effect on midgut infection rates, which for the different doses were 11% (n = 111), 12% (n = 116) and 21% (n = 120) compared to the control value of 12% (n = 119).

The addition of 100 μM 8-Br-cGMP to the bloodmeal at the second feed 48, 72 or 96 h post-infection significantly increased midgut infection rates of male *G. m. morsitans *from control values of 6% (n = 101), 14% (n = 99) and 22% (n = 97) to 73% (n = 105), 52% (n = 99) and 40% (n = 99) respectively (48 h: *p *< 0.001; 72 h: *p *< 0.001; 96 h: *p *= 0.005). The addition of 100 μM 8-Br-cGMP 120 h post-infection produced midgut infection rates of 10% (n = 83) which was not significantly different from the control value of 14% (n = 79; *p *= 0.391)

The continual addition of 100 μM 8-Br-cGMP had no significant effect on midgut infection rates (male: *p *= 0.600; female: *p *= 0.885) or on maturation (male: *p *= 0.373; female: *p *= 0.174) of midgut infections when compared to those receiving 100 μM 8-Br-cGMP as a single dose with the infective bloodmeal. In male flies midgut infection rates and TI rates for those fed 100 μM 8-Br-cGMP continually were 92% (n = 118) and 49% (n = 109) respectively compared to those which received only one dose of 100 μM 8-Br-cGMP which were 96% (n = 112) and 50% (n = 107) respectively. In female flies midgut infection rates and TI rates for those fed 100 μM 8-Br-cGMP continually were 95% (n = 83) and 24% (n = 79) compared to those which received only one dose of 100 μM 8-Br-cGMP which were 96% (n = 94) and 16% (n = 90) respectively.

The addition of 100 μM 8-Br-cAMP from the second feed after the infection with 100 μM 8-Br-cGMP had no significant effect on midgut infection rates (male: *p *= 0.603; female: *p *= 0.986) or on rates of maturation (male: *p *= 0.826; female: *p *= 0.491) of midgut infections when compared to those receiving 100 μM 8-Br-cGMP with the infective bloodmeal. In male flies midgut infection rates and TI rates for those fed 100 μM 8-Br-cAMP from the second bloodmeal were 91% (n = 58) and 51% (n = 53) compared to those which received one dose of 100 μM 8-Br-cGMP which were 96% (n = 74) and 49% (n = 71) respectively. In female flies midgut infection rates and TI rates for those fed 100 μM 8-Br-cAMP from the second feed were 99% (n = 87) and 16% (n = 89) compared to those receiving a single dose of 100 μM 8-Br-cGMP which were 99% (n = 89) and 20% (n = 87) respectively.

In the present work, results show that cAMP does not appear to be involved in either the establishment of midgut infections or the maturation of established midgut infections in tsetse. This contrasts with *in vitro *work where cAMP signalling was shown to be involved in transformation of the replicating long-slender form to the cell arrested short-stumpy [2]. However, we have shown that cGMP has a major effect on the susceptibility of tsetse flies to establishment of midgut trypanosome infections. There are two systems that the cyclic nucleotide could affect: the trypanosome and/or the tsetse fly. It has been suggested that treatment of malpighian tubules *in vitro *with cGMP modulates expression of anti-microbial peptides in *Drosophila *[5] and it has been reported that the antimicrobial peptide, attacin, is involved in trypanosome clearance from tsetse [6]. On the trypanosome side, although the genomes of *T. brucei *[7], *Trypanosoma cruzi *[8] and *Leishmania major *[9] did not show the presence of a typical guanylate cyclase, a cGMP dependent enzyme has been found in *Leishmania *[10] and a protein kinase has been shown to function through cGMP in *T. brucei *[11]. Previous studies found guanylate cyclase activity in *T. cruzi *[12] and it was suggested this activity was involved in cellular motility [13]. More recently guanylate cyclase activity has been found in *Leishmania donovani *[14].

8-Br-cGMP has been shown to induce both RNA and protein changes in cultured procyclic trypanosomes, indicating that cGMP signalling may be important in trypanosome biology [15]. We have shown that cGMP can "rescue" dying trypanosome infections up to four days after trypanosomes have entered the tsetse fly midgut; by contrast glutathione was only able to rescue such infections within two days post-infection [3], suggesting that the majority of trypanosomes normally die within two days of ingestion.

Unlike the continual feeding of glucosamine to infected tsetse (which decreased rates of trypanosome maturation [16]), in the present work continual feeding of 8-Br-cGMP had no effect on maturation rates of infected tsetse.

In conclusion the current work has shown that the guanylyl cyclic nucleotide, cGMP, increases susceptibility of tsetse flies to trypanosomes. Whether or not this effect works through the fly and/or the trypanosome is as yet unclear.

## Competing interests

The author(s) declare that they have no competing interests.

## Authors' contributions

ETM carried out the fly infection experiments and performed the statistical analysis. ETM, IM and SCW jointly planned the study, participated in its design and coordinated and drafted the manuscript. All authors have read and approved the final manuscript.

## References

[B1] Reuner B, Vassella E, Yutzy B, Boshart M (1997). Cell density triggers slender to stumpy differentiation of *Trypanosoma brucei *bloodstream forms in culture. Mol Biochem Parasitol.

[B2] Vassella E, Reuner B, Yutzy B, Boshart M (1997). Differentiation of African trypanosomes is controlled by a density sensing mechanism which signals cell cycle arrest via the cAMP pathway. J Cell Sci.

[B3] MacLeod ET, Maudlin I, Darby AC, Welburn SC (2007). Antioxidants promote establishment of trypanosome infections in tsetse. Parasitology.

[B4] MacLeod ET, Darby AC, Maudlin I, Welburn SC (2007). Factors affecting trypanosome maturation in tsetse flies. PLoS ONE.

[B5] Davies SA (2006). Signalling via cGMP: lessons from *Drosophila*. Cell Signal.

[B6] Hu C, Aksoy S (2006). Innate immune responses regulate trypanosome parasite infection of the tsetse fly *Glossina morsitans morsitans*. Mol Microbiol.

[B7] Berriman M, Ghedin E, Hertz-Fowler C, Blandin G, Renauld H, Bartholomeu DC, Lennard NJ, Caler E, Hamlin NE, Haas B, Böhme U, Hannick L, Aslett MA, Shallom J, Marcello L, Hou L, Wickstead B, Alsmark UC, Arrowsmith C, Atkin RJ, Barron AJ, Bringaud F, Brooks K, Carrington M, Cherevach I, Chillingworth TJ, Churcher C, Clark LN, Corton CH, Cronin A (2005). The genome of the African trypanosome *Trypanosoma brucei*. Science.

[B8] El-Sayed NM, Myler PJ, Bartholomeu DC, Nilsson D, Aggarwal G, Tran AN, Ghedin E, Worthey EA, Delcher AL, Blandin G, Westenberger SJ, Caler E, Cerqueira GC, Branche C, Haas B, Anupama A, Arner E, Aslund L, Attipoe P, Bontempi E, Bringaud F, Burton P, Cadag E, Campbell DA, Carrington M, Crabtree J, Darban H, da Silveira JF, de Jong P, Edwards K (2005). The genome sequence of *Trypanosoma cruzi*, etiologic agent of Chagas disease. Science.

[B9] Ivens AC, Peacock CS, Worthey EA, Murphy L, Aggarwal G, Berriman M, Sisk E, Rajandream MA, Adlem E, Aert R, Anupama A, Apostolou Z, Attipoe P, Bason N, Bauser C, Beck A, Beverley SM, Bianchettin G, Borzym K, Bothe G, Bruschi CV, Collins M, Cadag E, Ciarloni L, Clayton C, Coulson RM, Cronin A, Cruz AK, Davies RM, De Gaudenzi J (2005). The genome of the kinetoplastid parasite, *Leishmania major*. Science.

[B10] Géigel LF, Leon LL (2003). Cyclic 3'-5' guanosine monophosphate-dependent activity in *Leishmania amazonensis*. Mem Inst Oswaldo Cruz.

[B11] Shalaby T, Liniger M, Seebeck T (2001). The regulatory subunit of a cGMP-regulated protein kinase A of *Trypanosoma brucei*. Eur J Biochem.

[B12] Paveto C, Pereira C, Espinosa J, Montagna AE, Farber M, Esteva M, Flawiá MM, Torres HN (1995). The nitric oxide transduction pathway in *Trypanosoma cruzi*. J Biol Chem.

[B13] Pereira C, Paveto C, Espinosa J, Alonso G, Flawiá MM, Torres HN (1997). Control of *Trypanosoma cruzi *epimastigote motility through the nitric oxide pathway. J Eukaryot Microbiol.

[B14] Karmakar S, Ukil A, Mukherjee S, Das PK (2006). Regulation of guanylyl cyclase by intracellular Ca^2+ ^in relation to the infectivity of the protozoan parasite, *Leishmania donovani*. Int J Biochem Cell Biol.

[B15] Zeraia H (2006). Molecular analysis of insect stage *Trypanosoma brucei*. PhD Thesis.

[B16] Maudlin I, Welburn SC (1988). The role of lectins and trypanosome genotype in the maturation of midgut infections in *Glossina morsitans*. Trop Med Parasitol.

